# Supplementary material for: Catalytic influence of 16-s-16 gemini surfactants on the rate constant of histidine and ninhydrin
Source: R Soc Open Sci. 2020 Feb 5;7(2):191648. doi: 10.1098/rsos.191648 (PMC7062088; doi:10.1098/rsos.191648)
Supplement: Influence of pH and [His] on rate constant in graphical form. Absorbance vs. wavelength data, effect of [nin] on rate constant, and ratio data of rate constant in Tabular form [file rsos191648supp1.docx]

**Electronic Supplementary Material**

**Catalytic influence of 16-*s*-16 gemini surfactants on rate constant of histidine and ninhydrin**

**Dileep Kumar^1,2^ and Malik Abdul Rub^3^**

^1^Division of Computational Physics, Institute for Computational Science, Ton Duc Thang University, Ho Chi Minh City, Vietnam

^2^Faculty of Applied Sciences, Ton Duc Thang University, Ho Chi Minh City, Vietnam

^3^Chemistry Department, Faculty of Science, King Abdulaziz University, Jeddah-21589, Saudi Arabia

**Authors for correspondence:**

Dileep Kumar

email: dileepkumar@tdtu.edu.vn

**Figure S1.** Rate constant (*k*_ψ_) vs. pH on study of histidine and ninhydrin in surfactants: (A) 16-6-16, (B) 16-5-16, (C) 16-4-16. Reaction conditions: [His] = 1 x 10^-4^ mol dm^-3^, [ninhydrin] = 6.0 x 10^-3^ mol dm^-3^, [16-*s*-16] = 30 x 10^-5^ mol dm^-3^ and temp. = 343 K.

**Figure S2.** Rate constant (*k*_ψ_) vs. [His] on study of histidine and ninhydrin in surfactants: (A) 16-6-16, (B) 16-5-16, (C) 16-4-16. Reaction conditions: [ninhydrin] = 6.0 x 10^-3^ mol dm^-3^, [16-*s*-16] = 30 x 10^-5^ mol dm^-3^, temp. = 343 K and pH.

**Table S1.** Data of absorbance vs. wavelength (*λ*) on the study of histidine (1 x 10^-4^ mol dm^-3^) and ninhydrin (6.0 x 10^-3^ mol dm^-3^) in pure water and 16-*s*-16 (*s* = 4, 5, 6) surfactants (30 x 10^-5^ mol dm^-3^) at temperature (343 K) and pH (5.0).

| *λ* (nm) | Absorbance | | | |
| --- | --- | --- | --- | --- |
|  | Aqueous | 16-6-16 | 16-5-16 | 16-4-16 |
| 350 | 0.182 | 0.234 | 0.307 | 0.396 |
| 360 | 0.243 | 0.33 | 0.411 | 0.505 |
| 370 | 0.31 | 0.412 | 0.432 | 0.59 |
| 380 | 0.362 | 0.465 | 0.59 | 0.703 |
| 390 | 0.381 | 0.483 | 0.613 | 0.726 |
| 400 | 0.4 | 0.505 | 0.635 | 0.758 |
| 410 | 0.371 | 0.47 | 0.61 | 0.734 |
| 420 | 0.31 | 0.413 | 0.582 | 0.71 |
| 430 | 0.211 | 0.355 | 0.524 | 0.694 |
| 440 | 0.147 | 0.268 | 0.469 | 0.66 |
| 450 | 0.102 | 0.189 | 0.385 | 0.623 |
| 460 | 0.082 | 0.121 | 0.29 | 0.535 |
| 470 | 0.071 | 0.11 | 0.223 | 0.44 |
| 480 | 0.063 | 0.1 | 0.207 | 0.354 |
| 490 | 0.05 | 0.111 | 0.192 | 0.32 |
| 500 | 0.062 | 0.123 | 0.2 | 0.311 |
| 510 | 0.081 | 0.132 | 0.224 | 0.331 |
| 520 | 0.1 | 0.176 | 0.28 | 0.4 |
| 530 | 0.132 | 0.231 | 0.355 | 0.476 |
| 540 | 0.185 | 0.308 | 0.457 | 0.569 |
| 550 | 0.222 | 0.354 | 0.48 | 0.63 |
| 560 | 0.274 | 0.4 | 0.525 | 0.674 |
| 570 | 0.305 | 0.429 | 0.557 | 0.701 |
| 580 | 0.286 | 0.39 | 0.53 | 0.67 |
| 590 | 0.26 | 0.372 | 0.51 | 0.652 |
| 600 | 0.233 | 0.352 | 0.492 | 0.625 |
| 610 | 0.205 | 0.33 | 0.476 | 0.6 |
| 620 | 0.178 | 0.314 | 0.464 | 0.582 |
| 630 | 0.152 | 0.297 | 0.454 | 0.577 |
| 640 | 0.135 | 0.284 | 0.446 | 0.563 |
| 650 | 0.122 | 0.27 | 0.439 | 0.555 |

**Table S2.** Influence of ninhydrin concentration on rate constant (*k*_Ψ_) on the study on histidine and ninhydrin in 16-*s*-16 gemini surfactants (30 x 10^-5^ mol dm^-3^) at constant [His], temp. and pH.

| 10^3^ [Nin]  (mol dm^-3^) | 10^4^ *k*_ψ_ (s^-1^)  16-6-16 16-5-16 16-4-16 | | |
| --- | --- | --- | --- |
| 0 | 0 | 0 | 0 |
| 6.0 | 5.5 | 6.5 | 7.7 |
| 10.0 | 9.0 | 10.2 | 11.5 |
| 15.0 | 10.4 | 11.7 | 13.2 |
| 20.0 | 11.6 | 12.4 | 13.8 |
| 25.0 | 12.2 | 13.3 | 14.4 |
| 30.0 | 12.6 | 14 | 14.9 |
| 35.0 | 12.9 | 14.5 | 15.4 |
| 40.0 | 13.1 | 14.8 | 15.6 |

**Table S3.** Data of Determination of ratio (**$\frac{k_{\text{Ψ}}-k_{\text{Ψcal}}}{k_{\text{Ψ}}}$)** on the study of histidine (1.0 x 10^-4^ mol dm^-3^) and ninhydrin (6.0 x 10^-3^ mol dm^-3^) at temperature (343 K) and pH (5.0).

| 10^5^ [gemini]  (mol dm^-3^) | 16-6-16 | | | 16-5-16 | | | 16-4-16 | | |
| --- | --- | --- | --- | --- | --- | --- | --- | --- | --- |
|  | 10^4^ *k*_ψ_  (s^-1^) | 10^4^ *k*_ψcal_  (s^-1^) | **$\frac{k_{\text{Ψ}}-k_{\text{Ψcal}}}{k_{\text{Ψ}}}$** | 10^4^ *k*_ψ_  (s^-1^) | 10^4^ *k*_ψcal_  (s^-1^) | **$\frac{k_{\text{Ψ}}-k_{\text{Ψcal}}}{k_{\text{Ψ}}}$** | 10^4^ *k*_ψ_  (s^-1^) | 10^4^ *k*_ψcal_  (s^-1^) | **$\frac{k_{\text{Ψ}}-k_{\text{Ψcal}}}{k_{\text{Ψ}}}$** |
| 10.0 | 3.4 | 3.5 | -0.02941 | 4.2 | 4.3 | -0.02381 | 5.4 | 5.2 | 0.037037 |
| 20.0 | 4.7 | 4.6 | 0.021277 | 5.4 | 5.6 | -0.03704 | 6.8 | 6.5 | 0.044118 |
| 30.0 | 5.5 | 5.5 | 0 | 6.5 | 6.4 | 0.015385 | 7.7 | 8.0 | -0.03896 |
| 40.0 | 5.7 | 5.9 | -0.03509 | 6.7 | 6.5 | 0.029851 | 7.9 | 7.9 | 0 |
| 50.0 | 5.8 | 6.0 | -0.03448 | 6.8 | 6.6 | 0.029412 | 8.0 | 8.0 | 0 |
| 60.0 | 6.0 | 5.8 | 0.033333 | 6.9 | 7.1 | -0.02899 | 8.2 | 8.4 | -0.02439 |
| 80.0 | 6.2 | 6.3 | -0.01613 | 7.1 | 7.2 | -0.01408 | 8.3 | 8.7 | -0.04819 |
| 100.0 | 6.3 | 6.1 | 0.031746 | 7.2 | 7.2 | 0 | 8.5 | 8.3 | 0.023529 |
| 250.0 | 6.5 | 6.5 | 0 | 7.5 | 7.5 | 0 | 8.8 | 9.0 | -0.02273 |
| 400.0 | 6.8 | 7.0 | -0.02941 | 7.7 | 7.5 | 0.025974 | 9.2 | 9.2 | 0 |
| 600.0 | 7.2 | 7.1 | 0.013889 | 8.2 | 8.0 | 0.02439 | 10.0 | 9.8 | 0.02 |
